# WDR76 mediates obesity and hepatic steatosis via HRas destabilization

**DOI:** 10.1038/s41598-019-56211-6

**Published:** 2019-12-23

**Authors:** Jong-Chan Park, Woo-Jeong Jeong, Seol Hwa Seo, Kang-Yell Choi

**Affiliations:** 1 Translational Research Center for Protein Function Control, Yonsei University, Seoul, 03722 Korea; 2 Department of Biotechnology, College of Life Science and Biotechnology, Yonsei University, Seoul, 03722 Korea; 3 CK Biotechnology Inc., Rm 417, Engineering Research Park, 50 Yonsei Ro, Seodaemun-Gu, Seoul 03722 Korea

**Keywords:** Immunohistochemistry, Fats, Ubiquitylated proteins, Ubiquitin ligases

## Abstract

Ras/MAPK (mitogen active protein kinase) signaling plays contradictory roles in adipocyte differentiation and is tightly regulated during adipogenesis. However, mechanisms regulating adipocyte differentiation involving Ras protein stability regulation are unknown. Here, we show that WD40 repeat protein 76 (WDR76), a novel Ras regulating E3 linker protein, controls 3T3-L1 adipocyte differentiation through HRas stability regulation. The roles of WDR76 in obesity and metabolic regulation were characterized using a high-fat diet (HFD)-induced obesity model using *Wdr76*^*−/−*^ mice and liver-specific Wdr76 transgenic mice (*Wdr76*^*Li−TG*^). *Wdr76*^*−*/*−*^ mice are resistant to HFD-induced obesity, insulin resistance and hyperlipidemia with an increment of HRas levels. In contrast, *Wdr76*^*Li-TG*^ mice showed increased HFD-induced obesity, insulin resistance with reduced HRas levels. Our findings suggest that WDR76 controls HFD-induced obesity and hepatic steatosis via HRas destabilization. These data provide insights into the links between WDR76, HRas, and obesity.

## Introduction

Obesity and its metabolic complications have emerged as major public health problems in recent decades. Its occurrence leads to an increase in many pathological conditions, including insulin resistance, diabetes, nonalcoholic fatty liver disease (NAFLD), hyperlipidemia, hypertension, and cardiovascular disease^1–4^.

Adipose tissue and liver are crucial for whole-body insulin sensitivity and energy homeostasis^5,6^. The excessive accumulation of body fat in white adipose tissue (WAT) is the result of excessive growth, differentiation, and hypertrophy of adipocytes as fundamental processes of obesity^7^. Dysregulation of hepatic lipid metabolism is related to hepatic steatosis (fatty liver), which results in chronic insulin resistance^8^. During the progression of insulin resistance, insulin fails to suppress hepatic glucose production yet continues to drive excess lipid synthesis, leading to hyperglycemia, hyperlipidemia, and NAFLD^9^. Therefore, understanding the precise mechanism of metabolic regulation related to lipid metabolism provides new insight for the therapeutic approaches for the metabolic dysregulation.

Many signaling pathways, including the extracellular signal-regulated kinase (ERK), insulin, Wnt, TGF-β, and Notch pathways are involved in the regulation of adipogenesis and hepatic metabolism^10–18^. The Ras/ERK pathway plays important roles in adipogenesis via many functions from early to late adipocyte differentiation^14^. Ras/ERK pathway activation is required for mitotic clonal expansion in early adipocyte differentiation. However, constitutive activation of ERK blocks the terminal differentiation of adipocytes by phosphorylation of PPARγ^14^. Therefore, after proliferation, this pathway has to be down-regulated for terminal differentiation of adipocytes^14,15,19^. The Ras/ERK pathway also plays important roles in the processes that regulate hepatic metabolism in response to insulin^10,11^. Hepatic ERK2 deficiency promotes impairment of glucose metabolism and insulin resistance^11^. Additionally, when fed a high-fat diet (HFD), mice deficient in hepatic ERK2 have increased levels of triglycerides in the liver leading to the development of hepatic steatosis^11^. However, the effects of Ras protein stability regulation in adipogenesis and liver metabolism are not known.

Ras proteins regulate various cellular processes including proliferation, differentiation, and survival by the alternative binding states of guanosine triphosphate (GTP) and guanosine diphosphate (GDP) or subcellular localization, followed by control of its differential downstream effectors^20–24^. In addition, stability regulation of Ras, as an alternate approach for control of Ras activity, also plays important roles in pathophysiology^25–34^.

We recently identified WDR76, a component of E3 ubiquitin ligase complex, as one of the HRas binding proteins that mediates Ras degradation, thus functioning as a tumor suppressor in liver cancer and colorectal cancer^34,35^. As an E3 linker protein, WDR76 was found in the CUL4-DDB1 ubiquitin ligase complex^36^. This complex is known to be involved in the regulation of circadian rhythms^37^ and the suppression of hepatocarcinogenesis via polyubiquitination-dependent degradation of Ras^34^.

It is known that a wide variety of cellular processes including metabolic homeostasis are regulated by ubiquitination-dependent protein turnover^38^. E3 ubiquitin ligases and its interacting proteins, such as MKRN1^39^, WDTC1^40^, COP1^41^, and Fbxw7^42^, are also known to contribute to this process. However, the physiological roles of HRas protein stability regulation by WDR76 in HFD-induced obesity and hepatic steatosis are unknown.

We therefore investigated the roles of the Ras destabilizer WDR76 in preadipocyte differentiation using 3T3-L1 cells, and in obesity and hepatic steatosis using a HFD-induced obesity model. The HFD-fed *Wdr76*^*−/−*^ mice showed improved metabolic and physiological parameters, including decreased obesity, insulin resistance, and hyperlipidemia with increases of HRas levels compared with *Wdr76*^*+/+*^ mice. In contrast, liver-specific Wdr76 transgenic mice (*Wdr76*^*Li-TG*^) were characterized showing increased HFD-induced metabolic defects including obesity and insulin resistance with reduced HRas levels.

In this study, we identified the role of WDR76 in HRas destabilization related to adipocyte differentiation of the 3T3-L1 preadipocytes. Moreover, we further characterized the roles of WDR76 in HFD-induced obesity and hepatic steatosis, suggesting WDR76 as a potential target for the treatment of obesity and metabolic diseases related to HFD.

## Results

### WDR76 mediated adipocyte differentiation of 3T3-L1 cells via destabilization of HRas

**Figure 1:**
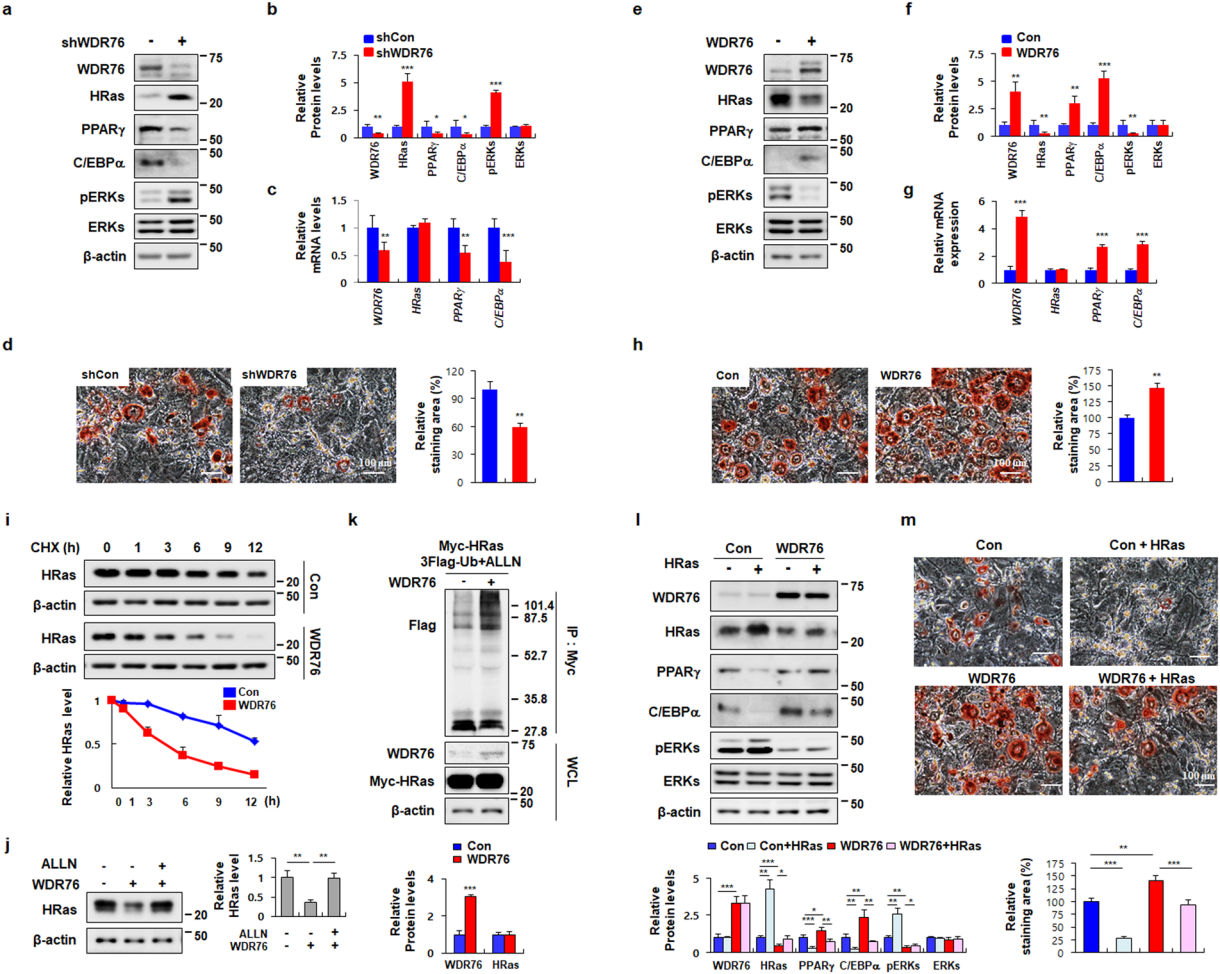
Effects of WDR76 on adipogenesis in 3T3-L1 cells. (**a**–**d**) 3T3-L1 cells infected with shCon or shWDR76 lentivirus were grown under MDI-induced differentiation condition. (**e**–**h**) The 3T3-L1 cells infected with control or WDR76 lentivirus were grown under MDI-induced differentiation conditions. (**a**,**e**) Immunoblot (IB) analyses were performed to detect WDR76, PPARγ, C/EBPα, HRas, pERKs, ERKs, and β-actin. (**b**,**f**) Quantification of IB analyses as determined by Image J. (**c**,**g**) The mRNA levels of *WDR76*, *PPAR*γ, *C/EBP*α, and *HRas* were measured by qRT-PCR using β*-actin* as an internal control. (**d**,**h**) Lipid droplets were stained using Oil Red O staining (ORO). Scale bars, 100 µm. Right panels show graphs presenting the relative area of ORO staining as determined by Image J. (**i**) The levels of HRas at indicated time points after CHX treatment were determined by IB, and were quantified with β-actin as a loading control. Results plotted on the lower are the amounts of HRas at each time point relative to the level at time 0. (**j**) The 3T3-L1 cells were infected with control or WDR76 lentivirus and then treated with *N*-acetyl-leucyl-leucyl-norleucinal (ALLN; 25 µg/mL) where indicated. (**k**) The 3T3-L1 cells were transfected with pLVX-IRES-Hyg-Myc-H-Ras, pCS4-3xFlag-Ub, and/or pLVX-IRES-Hyg-WDR76 and then treated with ALLN for 12 h. Whole-cell lysates (WCLs) were then immunoprecipitated with an anti-Myc antibody. The ubiquitin-conjugated proteins were detected by IB analyses. Lower panels show graphs for quantification of IB analyses. (**l**,**m**) The 3T3-L1 cells were infected with control or WDR76 with or without HRas lentivirus and grown under MDI-induced differentiation condition. WCLs were subjected to IB analyses using PPARγ, C/EBPα, WDR76, HRas, pERKs, ERKs or β-actin antibody. Lower panels show graphs for quantification of IB analyses (l). Lipid droplets were stained using ORO staining. Lower panels show graphs depicting the relative area of ORO staining as determined by Image J. Scale bars, 100 µm (m). All data are presented as the mean ± SD and representative results of at least three experiments are shown. *p < 0.05; **p < 0.01; ***p < 0.005, Student’s *t*-test.

To elucidate the role of WDR76 on HRas protein stability during adipogenesis, we examined the effect of the knockdown or overexpression of WDR76 in 3T3-L1 cells. In 3T3-L1 cells, knockdown of WDR76 increased HRas protein levels without changing HRas mRNA levels, resulting in the activation of ERKs (Fig. 1a–c). The role of WDR76 in inhibition of adipocyte differentiation was confirmed by decreases in both mRNA and protein levels of PPARγ and C/EBPα as well as reduction of Oil Red O (ORO) staining of 3T3-L1 cells (Fig. 1d) after WDR76 knockdown (Fig. 1a–d).

In contrast, WDR76 overexpression increased the PPARγ and C/EBPα mRNAs and protein levels with reduction of HRas protein levels and ERKs activation (Fig. 1e–g). ORO staining showed that lipid accumulation was increased by WDR76 overexpression (Fig. 1h). Degradation rates of HRas were also accelerated by WDR76 overexpression, as shown by measurements in the presence of the *de novo* protein synthesis inhibitor, cycloheximide (CHX) (Fig. 1i). The proteasome inhibitor *N*-acetyl-leucyl-leucyl-norleucinal (ALLN), reversed the WDR76-mediated HRas reduction (Fig. 1j). WDR76 overexpression increased HRas polyubiquitination (Fig. 1k and Supplementary Fig. 1). These results indicated that HRas degradation occurred through the polyubiquitination-dependent proteasomal machinery. The role of HRas stability regulation in adipocyte differentiation by WDR76 was confirmed by co-expression of WDR76 and HRas (Fig. 1l,m). HRas overexpression reduced the levels of both PPARγ and C/EBPα; whilst WDR76 overexpression increased PPARγ and C/EBPα expression with a decrease of HRas protein levels and ERKs phosphorylation (Fig. 1l).

HRas overexpression consistently blocked adipogenesis, but the effect was reversed by overexpression of WDR76 as shown by ORO staining (Fig. 1m). To examine the role of HRas in the WDR76-dependent adipocyte differentiation, we checked the effects of HRas knockdown on the WDR76-induced differentiation of 3T3-L1 cells (Supplementary Fig. 2a,b). The adipogenesis of 3T3-L1 cells monitored by ORO staining was increased by HRas knockdown or WDR76 overexpression together with induction of both PPARγ and C/EBPα (Supplementary Fig. 2a,b). The WDR76-induced adipogenesis was further increased by HRas knockdown (Supplement Fig. 2a,b).

Because WDR76 revealed a regulatory effect on preadipocyte differentiation, we examined expression levels of WDR76 and HRas during the preadipocyte differentiation of 3T3-L1 cells. WDR76 expression was increased up to day 2, and decreased thereafter. Contrarily, HRas level was gradually decreased up to day 8 after initiation of the differentiation (Supplementary Fig. 3a). In addition, we examined the levels of WDR76 and HRas in WAT and liver tissue of mice fed either normal chow diet (Chow) or HFD. WDR76 and HRas expression were increased and decreased, respectively, in both WAT and liver tissue of HFD-induced obese mice compared with the control mice fed a Chow diet (Supplementary Fig. 3b). Overall, WDR76 plays a role in adipocyte differentiation through destabilization of HRas in 3T3-L1 preadipocyte cells.

### WDR76 deficiency ameliorated HFD-induced obesity in mice

**Figure 2:**
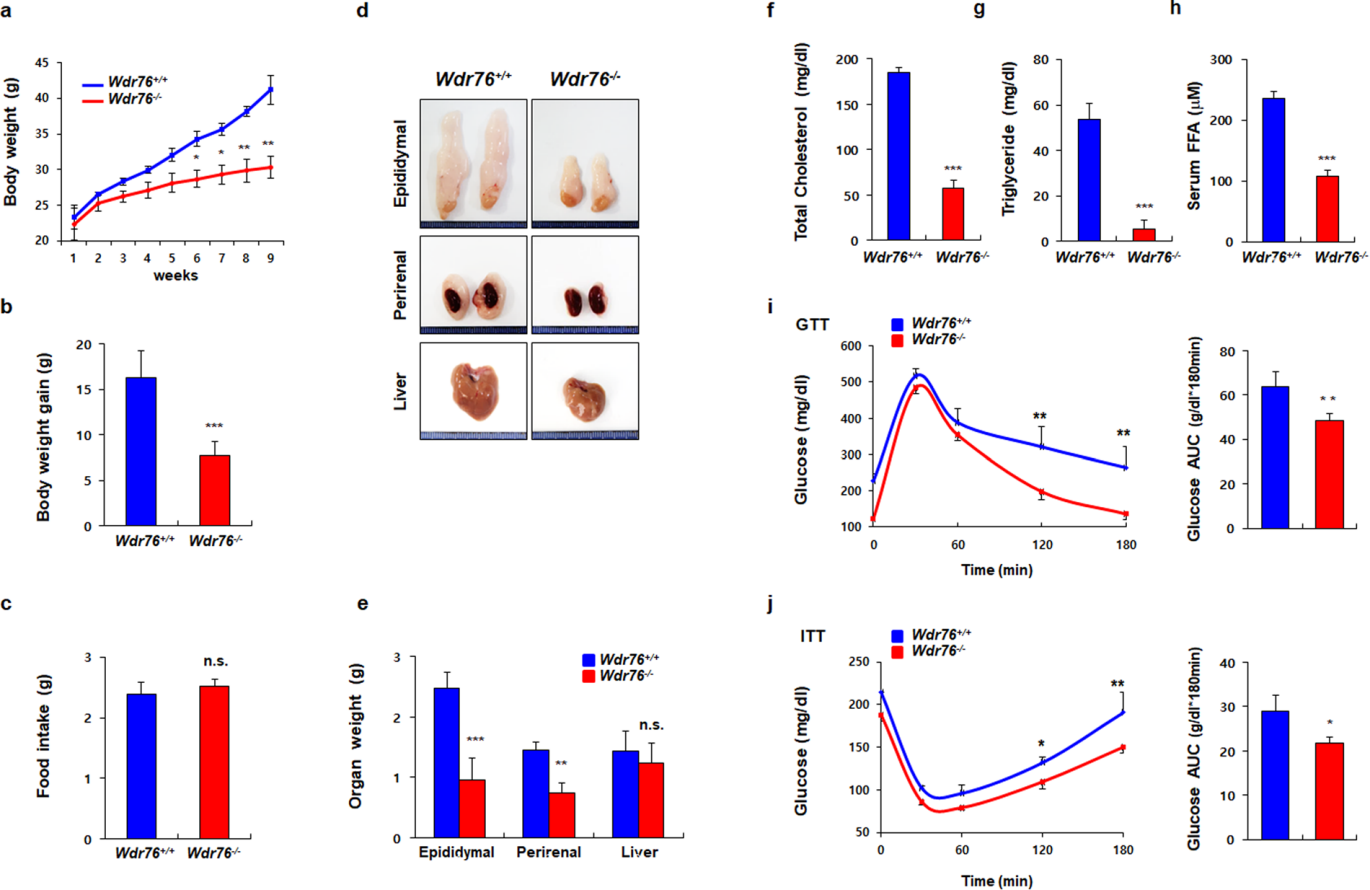
*Wdr76* knockout mice are resistant to high-fat diet (HFD)-induced obesity. (**a**–**c**) *Wdr76*^*+/+*^ and *Wdr76*^−*/*−^ mice were fed a HFD (60% calories from fat) for 9 weeks (*n* = 10 per group). Body weight (**a**), body weight gain (**b**), and food intake (**c**) were analyzed. (**d**,**e**) Photographed images and weights of various organs from *Wdr76*^*+/+*^ and *Wdr76*^−*/*−^ mice fed a HFD. (**f**–**h**) Serum levels of total cholesterol (**f**), triglyceride (**g**), and free fatty acids (**h**) in HFD-fed *Wdr76*^*+/+*^ and *Wdr76*^−*/*−^ mice were measured (*n* = 5 per group). (**i**,**j**) GTT, ITT and area under curve (AUC) of *Wdr76*^*+/+*^ and *Wdr76*^−*/*−^ mice (*n* = 5 per group). Data are presented as the mean ± SD. *p < 0.05; **p < 0.01; ***p < 0.005, Student’s *t*-test.

To assess whether there were overt phenotypic effects in the context of a HFD, we fed *Wdr76*^*+/+*^ and *Wdr76*^*−/−*^ mice with a HFD (60% kcal from fat) for 9 weeks, and monitored the body weights. The male *Wdr76*^*−/−*^ mice showed reduced body weights (41.2 ± 2.02 g vs 30.3 ± 1.54 g) (Fig. 2a,b) and the body weight reduction of the *Wdr76*^*−/−*^ mice was not attributed to differences in their food intake (Fig. 2c) or body length (Supplementary Fig. 4). The sizes and weights of epididymal, and perirenal fat tissues of *Wdr76*^*−/−*^ mice were all reduced compared to those of *Wdr76*^*+/+*^ mice (Fig. 2d,e). To identify the effects of WDR76 deficiency on metabolic disorders, we measured triglyceride (TG), total cholesterol (TC), and free fatty acid (FFA) levels in the serum of the HFD-fed *Wdr76*^*+/+*^ and *Wdr76*^*−/−*^ mice. Compared with *Wdr76*^*+/+*^ mice, *Wdr76*^*−/−*^ mice showed a decrease in the levels of TG, TC, and FFA in the serum (Fig. 2f–h).

Obesity is correlated with glucose intolerance and insulin resistance. Therefore, we next assessed the effects of WDR76 deficiency on glucose homeostasis and insulin sensitivity. *Wdr76*^*−/−*^ mice had improved glucose tolerance and insulin sensitivity (Fig. 2i,j), suggesting that WDR76 deficiency could reduce HFD-induced insulin resistance.

**Figure 3:**
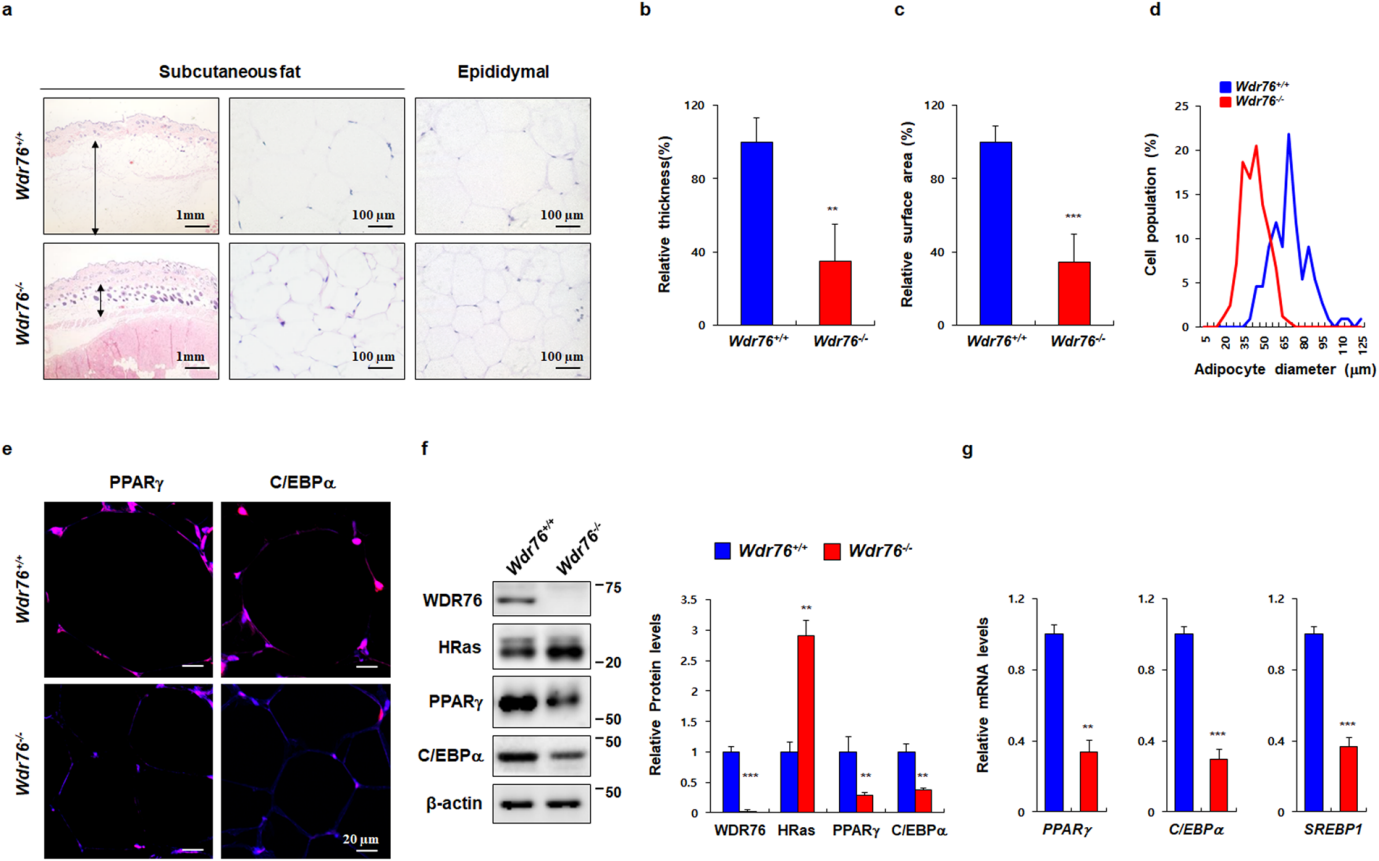
Effects of *Wdr76* knockout on adipose tissue of HFD-induced obese mice. (**a**–**d**) Subcutaneous fat and epididymal fat were stained by hematoxylin and eosin (H&E) (**a**) and analyzed (**b**–**d**); *n* = 5 per group). The relative thickness (**b**) and relative surface area (**c**) of subcutaneous fat was measured. Distribution of adipocyte size in epididymal white fat (WAT) was measured in three randomly chosen microscopic areas from five independent animals per group. (**d**) The diameters of epididymal white fat adipocytes were determined using the NIS element AR image program, and more than 300 adipocytes were examined for each group. (*n* = 5 per group). (**e**) Immunohistochemical (IHC) analysis of PPARγ and C/EBPα in epididymal WAT. Scale bars, 20 µm. (**f**,**g**) WDR76, PPARγ, C/EBPα, SREBP and HRas mRNA and protein levels were determined by IB (*n* = 3 per group) (**f**) and qRT-PCR (*n* = 5 per group). (**g**) Right panels show graphs for quantification of IB analyses. (**f**) Data are presented as the mean ± SD. Student’s *t*-test. **p < 0.01; ***p < 0.005.

The thickness and surface area of subcutaneous fat in HFD-fed *Wdr76*^*−/−*^ mice was smaller than that of HFD-fed *Wdr76*^*+/+*^ mice as determined by hematoxylin and eosin (H&E) staining (Fig. 3a–c). The diameters of epididymal white fat adipocytes were also reduced (Fig. 3d). Immunohistochemical (IHC) and western blotting analyses of epididymal WAT showed that protein levels of PPARγ and C/EBPα were lowered with an increase of HRas protein levels in *Wdr76*^*−/−*^ mice (Fig. 3e,f). The reduction of mRNA levels of the adipogenic transcription factors (PPARγ, C/EBPα, and SREBP1) in *Wdr76*^*−/−*^ mice were further confirmed by real-time quantitative polymerase chain reaction (qRT-PCR) (Fig. 3g).

### *Wdr76*^*−/−*^ mice had decreased hepatic steatosis

**Figure 4:**
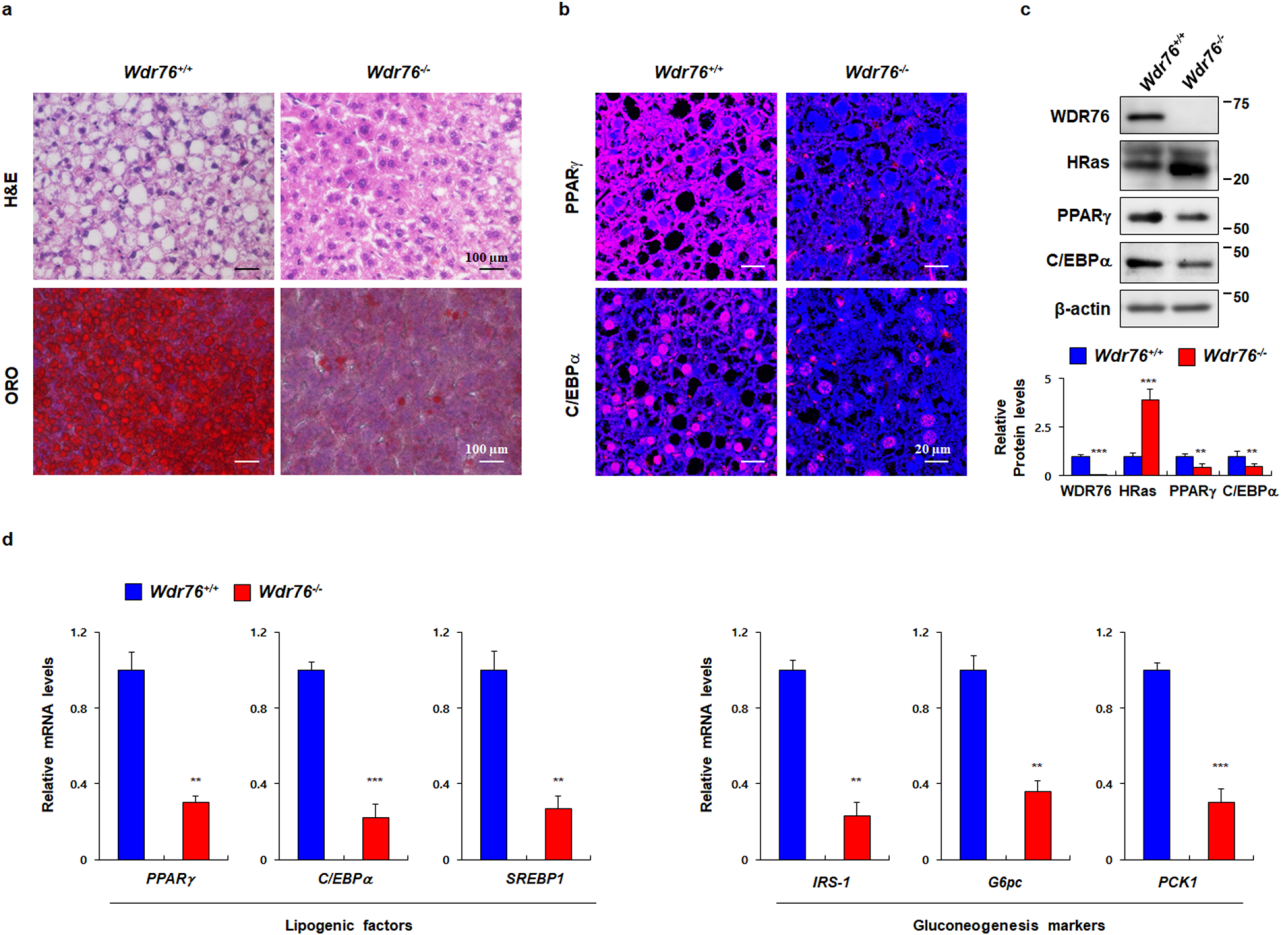
Effects of *Wdr76* knockout on livers of HFD-induced obese mice. (**a**) H&E and ORO staining of liver tissues from HFD-fed *Wdr76*^*+/+*^ and *Wdr76*^−*/*−^ mice, respectively. Scale bars, 100 µm. (**b**) IHC analyses of PPARγ and C/EBPα in liver. Scale bars, 20 µm. (**c**) IB analysis was performed from liver extracts of *Wdr76*^*+/+*^ and *Wdr76*^−*/*−^ mice to detect WDR76, HRas, PPARγ, C/EBPα and β-actin. Lower panels show graphs for quantification of IB analyses (*n* = 3 per group). (**d**) Hepatic gene expression profiles involved in metabolism from the livers of *Wdr76*^*+/+*^ and *Wdr76*^−*/*−^ mice as determined by qRT-PCR. Expression was normalized to *β-actin* expression (*n* = 5 per group). All data are presented as the mean ± SD. Student’s *t*-test. **p < 0.01; ***p < 0.005.

Obesity is closely related with hepatic steatosis in humans as well as in rodents. To further characterize the role of WDR76 in the hyperlipidemia of liver, histological characteristics of liver tissues of *Wdr76*^*−/−*^ mice were analyzed with ORO staining to characterize the possibility of fatty livers (Fig. 4a). Consistent with the lean phenotype in *Wdr76*^*−/−*^ mice, smaller lipid vesicles and decreased numbers of hepatocytes with lipid droplets were observed in the livers of *Wdr76*^*−/−*^ mice (Fig. 4a). Because hepatic PPARγ and C/EBPα proteins are known to play a role in the development and maintenance of hepatic steatosis, we assessed the expression levels of these in the liver tissues of HFD-fed *Wdr76*^*+/+*^ and *Wdr76*^*−/−*^ mice^43–45^. IHC analyses and western blotting of liver tissues revealed that expression levels of C/EBPα and PPARγ decreased with an increase of HRas protein in *Wdr76*^*−/ −*^ mice (Fig. 4b,c). To further investigate the role of WDR76 in hepatic steatosis, we analyzed the expression of key lipogenic and metabolic genes in the livers of HFD-fed *Wdr76*^*+/+*^ and *Wdr76*^*−/−*^ mice. The mRNA expression levels for both liver lipogenesis and gluconeogenesis markers were reduced in *Wdr76*^*−/−*^ mice (Fig. 4d). Taken together, the results showed that *Wdr76*^*−/−*^ mice had reduced hepatic steatosis.

### The liver-specific overexpression of WDR76 increased obesity and insulin resistance

**Figure 5:**
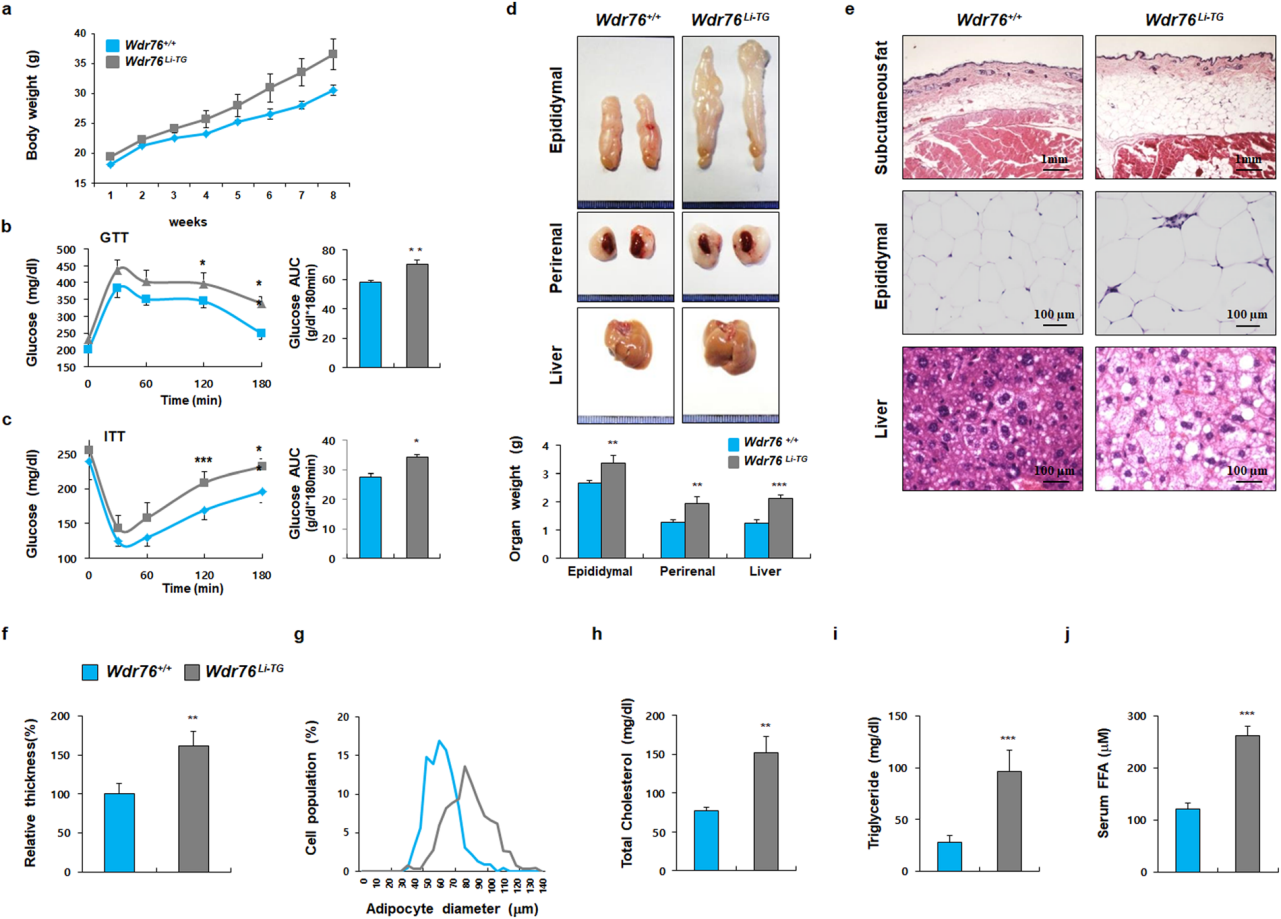
Liver-specific *Wdr76* overexpression mice were susceptible to HFD-induced obesity. (**a**) Body weight curves of *Wdr76*^*+/+*^ mice and *Wdr76*^*Li-TG*^ mice fed with a HFD (45% calories from fat) for 8 weeks (*n* = 10 per group). (**b**,**c**) GTT, ITT and AUC of HFD-fed *Wdr76*^*+/+*^ mice and *Wdr76*^*Li-TG*^ (*n* = 5 per group). (**d**) Photographed images and the weight of various organs from *Wdr76*^*+/+*^ and *Wdr76*^*Li-TG*^ mice fed a HFD. (**e**) H&E staining of Subcutaneous fat, epididymal fat, and liver tissues of *Wdr76*^*+/+*^ mice and *Wdr76*^*Li-TG*^ mice. (**f**) Relative thickness of subcutaneous fat was measured in three randomly chosen microscopic areas from five independent animals per group. (**g**) Distribution of adipocyte size in epididymal WAT was measured (n = 5 per group). The diameters of epididymal white fat adipocytes were determined using the NIS element AR image program, and more than 300 adipocytes were examined for each group. (**h**–**j**) Serum levels of total cholesterol (**h**), triglyceride (**i**), and free fatty acids (**j**) in HFD-fed *Wdr76*^*+/+*^ and *Wdr76*^*Li-TG*^ mice were measured (*n* = 5 per group). All data are presented as the mean ± SD. Student’s *t*-test. *p < 0.05; **p < 0.01; ***p < 0.005.

**Figure 6:**
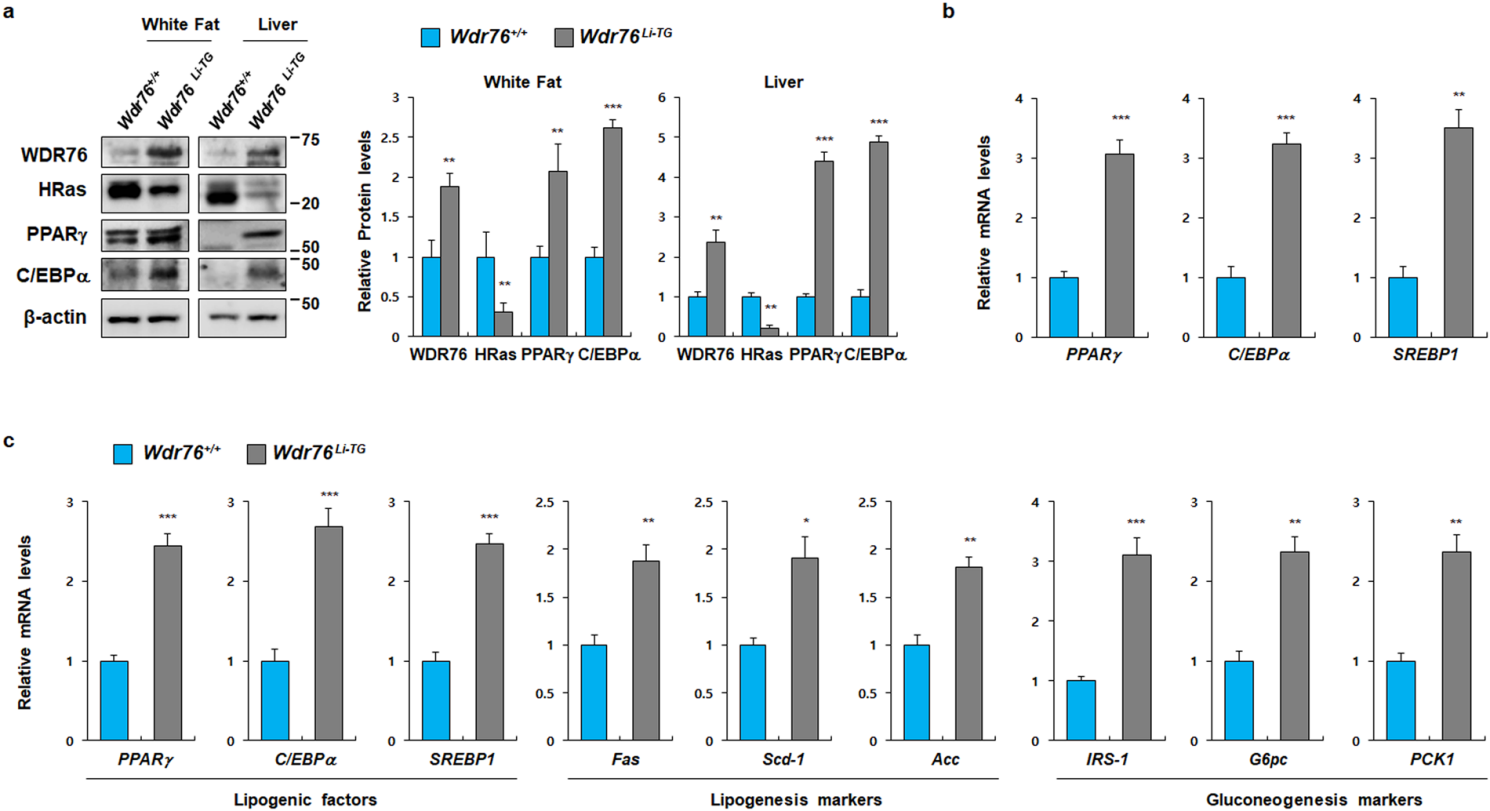
Effects of liver-specific *Wdr76* overexpression in HFD-induced obese mice. (**a**) IB analyses were performed from epididymal WAT and liver extracts of *Wdr76*^*+/+*^ and *Wdr76*^*Li-TG*^ mice to detect WDR76, HRas, PPARγ, C/EBPα and β-actin. Right panels show graphs for quantification of IB analyses (*n* = 3 per group). (**b**) Relative expressions of *PPAR*γ, *C/EBP*α, and *SREBP* in epididymal WAT of *Wdr76*^*+/+*^ and *Wdr76*^*Li-TG*^ mice (*n* = 5 per group). (**c**) Hepatic gene expression profile involved in metabolism from the liver of *Wdr76*^*+/+*^ and *Wdr76*^*Li-TG*^ mice as determined by qRT-PCR (*n* = 5 per group). Expression was normalized to *β-actin* expression. Data are presented as the mean ± SD. Student’s *t*-test. *p < 0.05; **p < 0.01; ***p < 0.005.

The liver plays a vital role in central metabolism by regulating key aspects of glucose and fatty acid regulation and storage^46^. Because *Wdr76*^*−/−*^ mice exhibited decreased hepatic steatosis, we examined the impact of WDR76 in the liver by using liver-specific *Wdr76* transgenic mice (*Wdr76*^*Li−TG*^). *Wdr76*^*Li−TG*^ mice were generated by crossing *Wdr76* conditional transgenic mice with albumin-Cre transgenic mice as previously described^34^. Based on the knockout mouse experiments, we hypothesized that *Wdr76*^*Li−TG*^ mice will be more sensitive to the HFD, resulting in a severe obesity phenotype compared with *Wdr76*^*+/+*^. Therefore, we fed *Wdr76*^*+/+*^ and *Wdr76*^*Li−TG*^ mice with a HFD (45% kcal from fat) in order to accurately quantify the effect of *WDR76* overexpression^47^. Although WDR76 was specifically overexpressed in the liver, *Wdr76*^*Li−TG*^ mice showed a severe obesity phenotype after HFD feeding when compared with *Wdr76*^*+/+*^ mice (Fig. 5a). *Wdr76*^*Li-TG*^ mice showed more glucose intolerant and insulin resistant phenotypes (Fig. 5b,c). Both sizes and weights of epididymal, perirenal WATs and liver were increased (Fig. 5d). Histological analyses of subcutaneous, epididymal adipose tissues and liver were performed to show that *Wdr76*^*Li-TG*^ mice had elevated fat depots with thicker subcutaneous fat, increased sizes of epididymal adipocytes and hepatic steatosis (Fig. 5e–g). Consistent with the increased fat mass by HFD feeding, TG, TC, and FFA levels in serum were increased in HFD-fed *Wdr76*^*Li-TG*^ mice when compared with those of *Wdr76*^*+/+*^ mice (Fig. 5h-j). We observed that WDR76 expression was increased in both WAT and liver tissue of HFD-fed *Wdr76*^*Li-TG*^ mice compared with *Wdr76*^*+/+*^ mice (Fig. 6a). As shown in Supplementary Fig. 3b, the level of WDR76 was increased in the WAT of HFD-fed mice. These data suggest that the increased level of WDR76 in WAT of HFD-fed *Wdr76*^*Li-TG*^ mice was affected by the severe obesity phenotype of *Wdr76*^*Li-TG*^ mice (Fig. 6a). The level of adipogenic markers, PPARγ and C/EBPα, was higher with decreased HRas levels in the liver and WAT of *Wdr76*^*Li-TG*^ mice (Fig. 6a). The mRNA expressions of *PPAR*γ, *C/EBP*α, and SREBP1 were increased in WAT of *Wdr76*^*Li-TG*^ mice (Fig. 6b). Consistently, hepatic gene expression profiles of lipogenesis and gluconeogenesis in the HFD-fed *Wdr76*^*Li-TG*^ mice were increased, suggesting that WDR76 promotes HFD-induced hepatic steatosis (Fig. 6c).

## Discussion

The Ras/ERK pathway has been known as an important signaling cascade in adipogenesis^14,48^. ERK has been reported to promote differentiation in the early stages of adipocyte differentiation and then ERK needs to be downregulated preceding adipocyte maturation^49,50^. HRas has been shown to block adipocyte differentiation, probably due to sustained activation of the ERK pathway^15,51^. These studies suggested that HRas protein might play roles in blocking adipocyte differentiation and should be down-regulated during adipogenesis. However, the regulatory mechanism of HRas during adipogenesis has been poorly explored.

Using a proteomics approach with tumor and non-tumor tissues from a human patient with hepatocellular carcinoma (HCC), the E3 linker protein, WDR76, was identified as an HRas binding protein, which promoted HRas degradation, thus functioning as a tumor suppressor in liver cancer^34^. However, the physiological roles of WDR76 regarding the stability regulation of Ras are unknown.

In this study, we found that WDR76-mediated Ras destabilization was directly related to adipocyte differentiation in 3T3-L1 cells. The effects of decreases and increases of HRas protein levels and ERK activity by overexpression and knockdown of WDR76, respectively, were verified in the regulation of 3T3-L1 preadipocyte cell differentiation. WDR76-mediated HRas degradation was regulated via polyubiquitination-dependent proteasomal degradation in 3T3-L1 cells. The role of HRas in blocking the adipogenesis was confirmed. HRas overexpression inhibited MDI-induced adipocyte differentiation of 3T3-L1 cells, which was consistent with the previous reports^51^. Overexpression of WDR76 reversed this effect through HRas degradation with the decrease of ERK activation. Consistently, HRas knockdown increased adipocyte differentiation of 3T3-L1 cells and these effects were further promoted by WDR76 overexpression. These results suggest that WDR76 mediates adipocyte differentiation of 3T3-L1 preadipocyte cells via HRas destabilization.

The role of WDR76 in adipocyte hypertrophy was confirmed by *in vivo* experiments using a HFD-induced obesity model. As we previously reported, whole-body *Wdr76* knockout mice had similar body weight and size, and no obvious developmental abnormalities^34^. When mice were fed with a HFD (60% kcal from fat), the *Wdr76*^−*/*−^ mice improved the risk factors of metabolic disorders induced by HFD, such as obesity, insulin resistance, size increments of the epididymal and perirenal fat tissues, hepatic steatosis as well as the increment of expressions of the lipogenic and gluconeogenic genes. These results suggest that WDR76 plays multiple pathophysiological roles related to metabolism and energy homeostasis. The impaired lipid storage in adipose tissue could lead to fat deposition in non-adipose tissues such as liver, heart, and muscle^52,53^. However, HFD-fed *Wdr76*^−*/*−^ mice showed decreased lipid storage in adipose tissue as results of size reduction of WATs but do not exhibit increased circulating TG levels or hepatic steatosis compared with *Wdr76*^*+/+*^ mice. The reduced accumulation of fat in the liver of the HFD-fed *Wdr76*^−*/*−^ mice is consistent with reduced expression of lipogenic genes, such as *PPAR*γ, *C/EBP*α, and *SREBP1* in the liver which play roles in the development of hepatic steatosis^54–57^. Because food intake or body length did not significantly alter between *Wdr76*^−*/*−^ and *Wdr76*^*+/+*^, it is possible that decreased body weight and hepatic steatosis phenotypes in HFD-fed *Wdr76*^−*/*−^ mice could be attributed to improved insulin sensitivity, altered glucose and lipid metabolism, or enhanced energy consumption *etc*. In addition, we also consider the possibility that deficiency of WDR76 in liver tissue could prevent fatty liver and improve lipid metabolism due to whole-body *Wdr76* knockout mice. Further investigations for lipid accumulation in muscle or brown adipose tissue, and the difference in energy expenditure compared with *Wdr76*^*+/+*^ mice will help to assess fat distribution of HFD-fed *Wdr76*^−*/*−^ mice.

The liver has a vital role in fatty acid synthesis and glucose and lipid metabolism. Fatty acid synthesized by the liver is converted to TG and transported to the blood^58^. Lipid accumulation in the liver is related to insulin resistance^59^. In addition, liver-specific gene regulation affects whole-body composition through lipid metabolism regulation^60,61^. To determine the effects of WDR76 in the liver, we introduced *Wdr76*^*Li-TG*^ mice with a HFD (45% kcal from fat) model. The differences in the H&E staining patterns of liver tissues and levels of TC, TG, and FFAs may be attributed to the differences in body weights of the control groups for *Wdr76*^*Li-TG*^ and *Wdr76*^−*/*−^ mice, respectively. A difference between the experimental conditions for the two mice groups is their dietary conditions; *Wdr76*^*+/+*^ and *Wdr76*^−*/*−^ mice fed a HFD (60% calories from fat) for 9 weeks, whereas *Wdr76*^*+/+*^ and *Wdr76*^*Li-TG*^ mice fed a HFD (45% calories from fat) for 8 weeks. Therefore, the observed phenotypic differences could likely be attributed to their differences in dietary conditions. The decrease in HRas protein levels by WDR76 overexpression *in vivo* was verified. HFD-fed *Wdr76*^*Li-TG*^ mice had increased HFD-related metabolic defects such as obesity, insulin resistance, and hyperlipidemia, with whole-body composition changes. These results supported the concept that the liver is one of the main systemic organs that regulate whole body glucose and lipid metabolism, and HRas stability regulation by WDR76 plays a role in metabolism regulation.

Consistent with our study, several studies reported the role of HRas in energy homeostasis. For example, adipose specific HRas transgenic mice showed reduced gonadal fat pad weight and reduced adipocyte size^62^. In addition, mice with oncogenic HRas mutation (*HRas*^*G12S/+*^) are resistant to the HFD-induced obesity^63^. Taken together, these results imply that the stability regulation of HRas by WDR76 is important in the maintenance of liver homeostasis.

There are multiple factors involved in the etiology of HCC^64,65^. Hepatic steatosis is one of the factors that can progress to cirrhosis and, subsequently to HCC^64^. We recently verified the tumor-suppressive role of WDR76, which functions via Ras degradation, by monitoring effects the diethylnitrosamine (DEN)-induced heptocarcinogenesis in *Wdr76*^−*/*−^ mice and the *HRas*^*G12V*^-driven liver carcinogenesis in *Wdr76*^*Li-TG*^ mice^34^. In the current study, however, we demonstrated that HFD-fed *Wdr76*^*Li-TG*^ mice had a severe obesity phenotype with increased hepatic steatosis which is a potential risk factor for HCC. Therefore, these results implicate that the stability regulation of HRas by WDR76 should be considered depending on pathophysiological conditions.

In summary, our data indicated that WDR76 plays a role as a positive regulator of diet-induced obesity and hepatic steatosis in mice through HRas destabilization. Considering a single E3 ligase may target multiple substrates, identifying the other potential targets for WDR76 will help to identify additional molecular mechanisms of WDR76. Moreover, further studies using adipose or liver tissue-specific *Wdr76* knockout mice and validating the role of WDR76 in adipogenesis *in vivo* will help to clarify the roles of WDR76 in metabolism and as a potential therapeutic target to control obesity-associated metabolic diseases.

## Methods

### Animals and dietary treatments

All animal experiments were performed in accordance with the Korean Food and Drug Administration guidelines. Protocols were reviewed and approved by the Institutional Animal Care and Use Committee (IACUC) of Yonsei University (IACUC-201711-652-01). The generation of *Wdr76*^−*/*−^ and *Wdr76*^*Li-TG*^ mice were previously described^34^. The 4–5-week-old *Wdr76*^*+/+*^ and *Wdr76*^−*/*−^ littermates (weight-matched) were fed with a HFD (60% calories from fat; Research Diet, D12492) for 9 weeks. The mice were weighed once a week for 9 weeks. The 4–5-week-old *Wdr76*^*+/+*^ and *Wdr76*^*Li-TG*^ littermates (weight-matched) were fed with a HFD (45% calories from fat; Research Diet, D12451) for 8 weeks. The mice were weighed once a week for 8 weeks. All mice were in the C57BL/6 background.

### Histological analysis and immunohistochemistry

For Hematoxylin and eosin (H&E) staining and immunohistochemistry (IHC), dissected tissues were fixed in 4% paraformaldehyde in PBS and embedded in paraffin and sectioned into 4 μm slices. Paraformaldehyde-fixed paraffin sections were deparaffinized using xylene and rehydrated in serially diluted in ethanol, and stained with H&E or used for IHC. Images of the H&E staining were recorded with a TE-2000U bright-field optical microscope (Nikon, Tokyo, Japan). The diameters of epididymal WAT were determined using the NIS element AR image program, and more than 300 adipocytes were examined for each group. For IHC analyses, the slides were autoclaved in retrieval buffer (10 mM sodium citrate buffer, pH 6.0; Sigma-Aldrich, St. Louis, MO, USA) for antigen retrieval. The sections were blocked in PBS containing 5% bovine serum albumin (BSA) and 1% normal goat serum (Vector Laboratories, CA) at room temperature for 1 h. The sections were incubated overnight at 4 °C with the following dilution of primary antibodies: PPARγ (Santa Cruz Biotechnology, sc-271392, 1:300) and C/EBPα (Cell Signaling Technology, #2295, 1:100). The slides were washed with PBS, incubated with Alexa Fluor 488- (Molecular Probes, A32731, 1:300) or Alexa Fluor 555-conjugated IgG secondary antibodies (Molecular Probes, A32732, 1:300) at room temperature for 1 h, and counterstained with DAPI (Roche Life Science, 10236276001, 1:5,000) for 10 min. The images were captured using a LSM700 META confocal microscope (Carl Zeiss).

### Cell culture and adipocyte differentiation

The 3T3-L1 preadipocytes were cultured as previously described^12,13^. The cells were maintained in Dulbecco’s Modified Eagle Medium (DMEM) with 10% (v/v) calf serum (BCS; Gibco), 100 µg/mL penicillin, and 100 µg/mL streptomycin in a 5% CO_2_ incubator at 37 °C. The cells were infected with lentivirus and adipocyte differentiation was induced after 48 h. For adipocyte differentiation, confluent cells were induced to differentiate in DMEM containing 10% fetal bovine serum (FBS; Gibco) and MDI (520 µM methylisobutylxanthine (IBMX; Sigma-Aldrich), 1 µM dexamethasone (Sigma-Aldrich) and 167 nM insulin(Gibco)). After 2 days, the medium was changed with DMEM containing 10% FBS and insulin. On day 4, the medium was replaced with DMEM containing 10% FBS and changed with fresh identical medium every 2 days for up to day 8. On day 8, cells were harvested for further analyses.

### Oil red O staining

For ORO staining, liver tissue samples were fixed using 4% paraformaldehyde in PBS of the liver, specimens were submerged in 15%, 20% and 30% sucrose solution per day. The samples were embedded in OCT compound (Tissue tek; Sakura Finetek USA) and sectioned into 20 μm slices. 3T3-L1 cells and liver tissues were washed with PBS and 70% isopropanol (Duksan Pure Chemicals), and stained with ORO solution (Sigma-Aldrich) at room temperature overnight. Samples were washed thoroughly with distilled water. Tissues were counterstained with Gill’s hematoxylin (Sigma-Aldrich). Images of the ORO staining were visualized with a bright field microscope (Nikon TE-2000U). To quantify the ORO staining intensities, image files were analyzed using ImageJ^66^. First, original images of staining were converted into RGB images, and the red monochromatic image was used to calculate the area of the staining. “Threshold” tool in the “Adjust” box in the “Image” menu was used to adjust threshold manually. And then staining positive area was obtained by clicking “Measure” button under the “Analyze” menu.

### Plasmids and reagents

The following plasmids have been described previously: pLKO.1-shHRas, pLVX-IRES-Hyg control, pLVX-IRES-Hyg-HRas, pLVX-IRES-Hyg-Myc-HRas, psPAX2, pMD2.G.^31^, pLVX-IRES-Hyg-WDR76, pLKO.1-shWDR76^34^, pCS4-3xFlag-Ub^25^, Plasmid transfections were performed using Lipofectamine (Invitrogen, Carlsbad, CA) according to the manufacturer’s instructions. The N-acetyl-Leu-Leu-Nle-CHO (ALLN) and cycloheximide (CHX) were purchased from Sigma-Aldrich and were administered at 25 and 50 μg/ml, respectively.

### RNA isolation and real-time quantitative PCR

Total RNA was extracted using the TRIzol reagent (Invitrogen, Carlsbad, CA, USA). Reverse transcription was performed with MLV reverse transcriptase (Invitrogen) using 2 μg of total RNA. Synthesized cDNA was diluted to a concentration of 100 ng/µL. Quantitative PCR analyses were performed using a Rotor-gene Q Real-time PCR cycler (Qiagen, Valencia, CA, USA) using SYBR green reagent (Qiagen) at 95 °C for 10 min followed by 40 cycles at 95 °C for 5 s and 60 °C for 15 s. Relative quantitation of mRNA levels of *WDR76*, *HRas*, *PPAR*γ, *C/EBP*α, *SREBP1*, *IRS-1*, *G6pc*, *PCK1*, *FAS*, *SCD-1* and *ACC* were estimated using the comparative Ct method (∆∆Ct). All mRNA values were normalized with respect to *β-actin*. PCR primer sequences are listed in Supplementary Table 1.

### Immunoblot analysis

Immunoblotting was performed as described previously^25,31^. Briefly, cells were washed in ice-cold PBS and lysed with radioimmunoprecipitation assay (RIPA) buffer (Millipore, Billerica, MA). The Bradford protein assay is used to measure the concentration of total protein in a sample. Equal amounts of protein (20–50 μg/lane) were loaded onto a 12% SDS-PAGE gel, resolved by electrophoresis, and subsequently transferred to nitrocellulose membranes. Membranes were blocked with 7.5% skim milk in PBS for 1 h at room temperature, and then blotted overnight with the appropriate antibody at 4 °C. The following antibodies and dilutions were used: HRas (Santa Cruz Biotechnology, sc-520, 1:1000), PPARγ (Abcam, ab19481, 1:1,000), C/EBPα (Cell Signaling Technology, #2295, 1:1000), WDR76 (Lab made^34^, 1:1,000), Myc (Cell Signaling Technology, #2276 S, 1:3000), p-ERK (Cell Signaling Technology, #9101 S, 1:1000), FLAG (Sigma-Aldrich, F7425, 1:3000), β-actin (Santa Cruz Biotechnology, sc-47778, 1:3000) and total ERKs (Santa Cruz Biotechnology, sc-514302, 1:5000). Horseradish peroxidase-conjugated anti-mouse (Cell Signaling Technology, #7076, 1:5000) and anti-rabbit (Bio-Rad, #1706515, 1:5000) secondary antibodies were used for 1 h at room temperature. The band signals were acquired with a LAS-4000 LCD camera coupled to MultiGauge software (Fuji). Quantification of band intensities for each blot was performed using Image J. The intensity of each band was normalized with the intensity of β-actin. Uncropped blots are available in Supplementary Figs. 5 and 6.

### Ubiquitination assay

For the ubiquitination assay, cells were washed in ice-cold PBS and lysed with RIPA buffer. Ten millimolar N-ethylmaleimide (NEM; Sigma-Aldrich) was subsequently added to the RIPA buffer for the ubiquitination assays. The lysates were incubated with the indicated antibodies and protein A/G agarose at 4 °C for 12 h, and the immunoprecipitated beads were washed three times in RIPA buffer. The ubiquitin-conjugated proteins were resolved by SDS-PAGE, and detected by IB analyses.

### Virus production and viral packaging

Lentiviral plasmid was co-transfected with the packaging plasmids, psPAX2 and pMD2.G into HEK 293 T cells using a ratio of 2:1.2:0.8, respectively. Lentivirus-containing media were collected twice every 24 h and filtered through 0.45 µm pore filters (EMB Millipore). During ultraconcentration of the virus in two steps, FBS was removed; samples were filtered through Centricon filters (EMB Millipore) with a 100 K cut-off, and then subjected to ultracentrifugation at 100,000 × *g* for 2 h at 4 °C. Concentrated lentivirus was reconstituted in PBS, prepared as 10 µL aliquots, and stored at −80 °C.

### Glucose tolerance test and insulin tolerance test

For glucose tolerance tests (GTTs) or insulin tolerance tests (ITTs), mice were intraperitoneally injected with D-glucose (1.5 g/kg body weight) after overnight starvation or human insulin (0.75 units/kg body weight) after 4 h starvation, respectively. Tail blood was drawn at indicated time intervals, and blood glucose level was measured with a One Touch Ultra glucometer (LifeScan). Area under the curve (AUC) for the GTT or ITT was calculated by GraphPad Prism program.

### Blood chemistry

Total blood samples from mice were collected by cardiac puncture. The blood was clotted for 30 min and then centrifuged for 10 min at 1,000 × g to obtain the supernatant. The supernatant was measured for metabolic parameters. Plasma free fatty acid (FFA) concentrations in plasma were measured with an ELISA kit (Cayman Chemical, Ann Arbor, MI, USA). Serum total cholesterol and triglycerides were measured using FUJI DRI-CHEM slides (Fuji, Japan).

### Statistical analysis

The results were expressed as mean ± standard deviation (s.d.) values of at least triplicate experiments. Data are expressed as the mean ± SD. Statistical analyses were performed using the Student’s t-test. Asterisks denote statistically significant differences (n.s., not significant; *p < 0.05; **p < 0.01; ***p < 0.005).

## Supplementary information

Supplementary Information
