# Supplementary material for: WDR76 mediates obesity and hepatic steatosis via HRas destabilization
Source: Sci Rep. 2019 Dec 23;9:19676. doi: 10.1038/s41598-019-56211-6 (PMC6927951; doi:10.1038/s41598-019-56211-6)
Supplement: Supplementary file 1 — Supplementary Information [file 41598_2019_56211_MOESM1_ESM.docx]

**Supplementary information**

**WDR76 mediates obesity and hepatic steatosis via HRas destabilization**

Jong-Chan Park^1, 2, +^, Woo-Jeong Jeong^1, 2, +^, Seol Hwa Seo^1, 2^ & Kang-Yell Choi^1, 2, 3,^ *

^1^Translational Research Center for Protein Function Control, Yonsei University, Seoul, 03722, Korea

^2^Department of Biotechnology, College of Life Science and Biotechnology, Yonsei University, Seoul, 03722, Korea

^3^CK Biotechnology Inc., Rm 417, Engineering Research Park, 50 Yonsei Ro, Seodaemun-Gu, Seoul 03722, Korea

*Correspondence: [kychoi@yonsei.ac.kr](mailto:kychoi@yonsei.ac.kr)

^+^These authors contributed equally to this work

**Supplementary Figure 1. WDR76-mediated HRas polyubiquitination in 3T3-L1 cells.** The 3T3-L1 cells were transfected with pLVX-IRES-Hyg-Myc-H-Ras, pCS4-3xFlag-Ub, and/or pLVX-IRES-Hyg-WDR76 and then treated with ALLN for 12 h. Whole-cell lysates (WCLs) were immunoprecipitated with an anti-Myc antibody and then the ubiquitin-conjugated HRas were blotted with anti-Myc antibody. IB analyses were performed from WCL to detect WDR76, HRas and β-actin. Lower panels show graphs for quantification of IB analyses. Data are presented as the mean ± SD and representative results of at least three experiments are shown. ^***^*p*<0.005, Student’s *t*-test.

**Supplementary Figure 2. Effects of WDR76 on adipocyte differentiation in the absence of HRas. (a, b)** The 3T3-L1 cells were infected with control or WDR76 lentivirus with or without shHRas lentivirus, and grown under MDI-induced differentiation condition. WCLs were subjected to IB analyses using PPARγ, C/EBPα, WDR76, HRas, pERKs, ERKs, or β-actin antibodies. Lower panels show graphs for quantification of IB analyses (a). Lipid droplets were stained using ORO staining. Lower panels show graphs depicting the relative area of ORO staining as determined by Image J (*n* = 3). Scale bars, 100 µm (b). Data are presented as the mean ± SD. ^*^*p*<0.05; ^**^*p*<0.01; ^***^*p*<0.005, Student’s *t*-test.

**Supplementary Figure 3. Expression profiles of WDR76 and HRas. (a)** 3T3-L1 cells differentiation were induced by standard MDI medium condition and cells were harvested at each indicated time point. Whole cell lysates were subjected to IB analyses by using anti-WDR76, -HRas or -β-actin antibody. **(b)** WDR76 and HRas proteins were detected using lysates from white fat and liver tissue of mice fed a normal diet (Chow) or a HFD (45% kcal from fat). Right panels show graphs for quantification of IB analyses. All data are presented as the mean ± SD and representative results of at least three experiments are shown. ^*^*p*<0.05; ^**^*p*<0.01; ^***^*p*<0.005, Student’s *t*-test.

**Supplementary Figure 4. Body length comparison of HFD-induced *Wdr76^+/+^* and *Wdr76^-/-^* mice.** Photographed images of HFD-fed (60% calories from fat) *Wdr76^+/+^* and *Wdr76^-/-^* mice.

**Supplementary Figure 5. Uncropped blots for Figure 1**

**Supplementary Figure 6. Uncropped blots for Figure 3f, 4c, 6a and Supplementary Figure 1, 2a, 3a, 3b.**

**Supplementary Table 1.** **Primer sequences**
